# INEQUALITY IN COMMUNITY HEALTH SERVICES USE IN ISRAEL: A COMPARISON BETWEEN ELDER JEWS AND ARABS

**DOI:** 10.1093/geroni/igz038.3169

**Published:** 2019-11-08

**Authors:** Ariela Lowenstein, Sigal Pearl Naim

**Affiliations:** 1 University of Haifa, Haifa, Israel; 2 Yezreel Valley Academic College, Yezreel Valley, Israel

## Abstract

Population aging is an important social and public health issue globally. However, increase in longevity causes physical frailty and disability for many elders, which might lead to independence loss and impact quality of life. This increases health services usage and leads to higher costs of medical treatments. Data show that higher socio-economic status and accessibility to health services might reduce inequality in service use and impact mortality rates and quality of life. Also, that improved socio-economic status and population accessibility to Health services may stem from inner health system factors, as well as those related to the patients. Among minorities lower usage of formal professional services, including health services, are often related to cultural differences and many times to lower technological level, which are not considered by service providing organizations. Thus, lack of attention to service using minorities’ needs may cause a gap between potential consumers to services use. Israel is a multi-cultural society with mixed population of Jews and Arabs. Currently, Arabs comprise 20.9% and Jews 74.3%. However, the rate of disabled Jews is 16% compared to 31% among older Arabs.

